# Social Comparisons, Self-Conceptions, and Attributions: Assessing the Self-Related Contingencies in Leader-Member Exchange Relationships

**DOI:** 10.1007/s10869-019-09628-9

**Published:** 2019-04-23

**Authors:** Émilie Lapointe, Christian Vandenberghe, Ahmed K. Ben Ayed, Gary Schwarz, Michel Tremblay, Denis Chenevert

**Affiliations:** 1 Nottingham University Business School China, The University of Nottingham Ningbo China, Ningbo, China; 2 HEC Montréal, Montréal, Canada; 3 McGill University, Montréal, Canada; 4 SOAS, University of London, London, England

**Keywords:** leader-member exchange, social comparison, self-concept, organizational commitment, work performance

## Abstract

Research on leader-member exchange (LMX) has demonstrated that, in addition to the value of LMX as an indicator of quality relationships with leaders, employees also evaluate how their relationship with the leader compares to other employees’ relationship with the leader. This finding led to the emergence of LMX social comparison (LMXSC). This study examines how LMX vs. LMXSC relates to work outcomes and considers the employee and perceived supervisor self-concept levels as moderators. We posit that LMX predicts work performance through increased organizational commitment. We further suggest that the relational and collective levels of the self-concept act as contingencies of the relationships among LMX, LMXSC, commitment, and performance. A sample of 250 employee-supervisor dyads was used to test the hypotheses. LMX predicted commitment and, indirectly, performance. The employee and perceived supervisor relational self-concepts acted as moderators of LMXSC, and the perceived supervisor collective self-concept acted as a moderator of LMX and LMXSC. However, not all moderation hypotheses were supported. Unexpected moderating effects involving the employee and perceived supervisor individual self-concepts, as well as main effects, were also uncovered. This study helps differentiate LMX from LMXSC and understand the role of self-conceptions, including self-conceptions attributed by employees to the leader, in leader-member relationships.

Rooted in social exchange theory (Blau, 1964), leader-member exchange (LMX), which reflects the quality of one’s relationship with the leader, has been widely studied in recent years. Research has shown that followers reciprocate the benefits associated with LMX (e.g., support, trust, valued resources, and challenging job assignments) through positive attitudes and behaviors (Dulebohn, Bommer, Liden, Brouer, & Ferris, 2012; Rockstuhl, Dulebohn, Ang, & Shore, 2012). Recently, LMX researchers have turned their interest to the social aspects of leader-member relationships (e.g., Henderson, Liden, Glibkowski, & Chaudhry, 2009; Vidyarthi, Liden, Anand, Erdogan, & Ghosh, 2010). By supplementing social exchange theory (Blau, 1964) with social comparison theory (Festinger, 1954), Vidyarthi et al. (2010) demonstrated that one’s relative LMX standing in the workgroup, a construct termed “LMX social comparison” (LMXSC), influences job performance and citizenship behavior beyond LMX and objective LMX standing. This finding suggests that in addition to the value of LMX as an indicator of social exchange relationships with leaders, employees are also sensitive to how the quality of their relationship with the leader compares to the quality of other employees’ relationship with the leader (i.e., LMXSC) (Vidyarthi et al., 2010; Wood, 1996).

In addition to Vidyarthi et al.’s (2010) study, little research has examined how LMXSC, i.e., social comparison processes, adds to the understanding of leader-member relationships beyond social exchange-based mechanisms. Of the utmost importance, research has yet to uncover the conditions in which LMX, as a social exchange-based variable, and LMXSC, as a social comparison-based variable, best exert their effects on work outcomes. Filling these gaps is critical not only to better differentiate LMX and LMXSC but also to gain a fuller understanding of leader-member relationships and the contingences in which leader-member relationship variables are more (vs. less) likely to influence work outcomes. This endeavor is also warranted from a practitioner’s perspective as it should help them to understand which aspects of leader-member relationships are critical to generate positive attitudes and behaviors and to provide insights into how to maximize positive outcomes.

From this perspective, the first goal of this study is to examine the relations of LMX and LMXSC to affective organizational commitment (hereafter, organizational commitment; Meyer & Allen, 1991) and, indirectly, to individual work performance dimensions (i.e., task proficiency and task adaptivity and proactivity; Griffin, Neal, & Parker, 2007). We focus on organizational commitment as a key mediator because it is a central outcome of LMX (Dulebohn et al., 2012; Rockstuhl et al., 2012), is reflective of a social exchange process and is known to foster pro-organizational behaviors (Lavelle, Rupp, & Brockner, 2007; Meyer & Allen, 1991; Meyer, Stanley, Herscovitch, & Topolnytsky, 2002). Because organizational commitment operates according to social exchange principles (van Knippenberg & Sleebos, 2006), the LMX-commitment relationship is expected to be stronger than the LMXSC-commitment relationship. However, favorable contingencies, as we argue below, would make LMXSC a relevant predictor of organizational commitment and, indirectly, of performance.

The second, related goal of this study is to investigate the boundary conditions of the relationships of LMX vs. LMXSC with organizational commitment and work performance. We specifically examine how key self-concept variables (i.e., employee and perceived supervisor individual, relational, and collective self-concept levels; Brewer & Gardner, 1996) moderate these relationships. This investigation has the potential to increase our understanding of how social exchange (i.e., LMX) and social comparison (i.e., LMXSC) processes are shaped by employees’ self-conceptions and perceptions of their supervisor’s self-conceptions. We primarily build on the research on attributions (Martinko, Harvey, & Douglas, 2007) to discuss the effect of perceived supervisor self-concept levels, an area that has not been fully explored in LMX research, and we argue that self-concept variables are involved in distinct interactions with LMX and LMXSC. As such, this study offers a comprehensive examination of the connections between self-conceptions and leader-member relationship variables. In the next sections, we introduce and discuss the hypothesized effects of LMX and LMXSC on commitment and, indirectly, on performance. We then discuss the self-concept variables and their hypothesized effects.

## Theoretical Background and Hypothesis Development

### LMX, LMXSC, Organizational Commitment, and Performance

LMX is based on a social exchange mechanism. Social exchange theory defines how two or more parties come to exchange resources, how series of exchanges proceed, and how exchanges impact the relationship between the parties involved (Cropanzano, Anthony, Daniels, & Hall, 2017). It stipulates that when one party treats another party favorably and is perceived as doing so, the receiving party will pay back the giver, as the norm of reciprocity prescribes (Gouldner, 1960). Social exchange theory also states that over time, reciprocal exchanges contribute to transform an economic exchange into a high-quality relationship characterized by socioemotional investments and open-ended obligations (Cropanzano & Mitchell, 2005; Cropanzano et al., 2017). As such, a high LMX prompts employees to reciprocate the favorable treatment received from the supervisor through positive attitudes and behavior (Dulebohn et al., 2012).

Because of its roots in social exchange principles and as supervisors act as agents of the organization, LMX should lead to organizational commitment. Indeed, employees generally view the supervisor as a representative of the organization who promotes the organization’s goals and steers employees’ behaviors on behalf of the organization (Eisenberger, Stinglhamber, Vandenberghe, Sucharski, & Rhoades, 2002). Thus, a favorable leader-member relationship characterized by support, loyalty, and trust (i.e., high LMX) reflects how well employees feel that they are being treated by the organization as exemplified by the supervisor. This should prompt employees to reciprocate the favorable treatment that they received, as exemplified by high LMX, through stronger organizational commitment, i.e., a stronger emotional attachment to, identification with, and involvement in the organization (Meyer & Allen, 1991).

Because organizational commitment indicates a high-quality, social exchange-based relationship with the organization (van Knippenberg & Sleebos, 2006) and acts as a conduit for reciprocal behaviors targeted at the organization (Lavelle et al., 2007; Meyer & Allen, 1991), it should also mediate the relationship between LMX and work performance. This idea is supported by prior meta-analytical reviews that report a significant relationship between organizational commitment and performance (Cooper-Hakim & Viswesvaran, 2005; Meyer et al., 2002; Riketta, 2002) and recent studies that show that organizational commitment mediates the relationships between positive leadership constructs (e.g., servant leadership, authentic leadership, and leader behavioral integrity; Lapointe & Vandenberghe, 2018; Leroy, Palanski, & Simons, 2012) and job performance. Therefore, we propose the following hypothesis:

- *Hypothesis 1*: LMX will be positively related to work role performance through increased organizational commitment.

LMXSC’s focus differs from LMX’s focus. It does not capture the intrinsic quality of the relationship with the supervisor per se. Instead, it illustrates social comparison processes (Vidyarthi et al., 2010; Wood, 1996). Social comparison theory (Buunk & Gibbons, 2007; Festinger, 1954; Wood, 1996) posits that individuals possess a universal tendency to compare themselves to other people. They engage in social comparison processes to evaluate their own capabilities and gain self-relevant knowledge, which they then use to form their attitudes and behaviors. Favorable social comparisons foster self-enhancement and positive responses, while unfavorable social comparisons make people feel worse about themselves and react less positively (e.g., Lyubomirsky & Ross, 1997; Zell & Alicke, 2009). As leaders differentiate their treatment of followers (Graen & Uhl-Bien, 1995) and as followers are typically part of larger workgroups, individuals scrutinize the relationships of coworkers to the leader to determine the relative standing of their own relationship with the leader (Greenberg, Ashton-James, & Ashkanasy, 2007; Vidyarthi et al., 2010). LMXSC thus enables employees to perform a socially grounded calibration of the quality of their relationship with the leader.^1^ When employees perceive this relationship to be better off than other employees’ relationship with the leader (i.e., view themselves in a comparatively favorable situation), they should display more positive reactions (Vidyarthi et al., 2010).

Unlike LMX, we expect that LMXSC only unleashes its potential as a predictor of organizational commitment and performance under certain contingencies. Specifically, we argue that the levels of the self-concept, as defining oneself or as perceived to define one’s supervisor, provide cognitive frameworks that may guide (i.e., enhance or weaken) the effects of LMXSC on commitment and performance. This is because the self-concept variables likely shape the meaning and value of the social comparison processes for individuals. Similarly, self-conceptions may come into play as frameworks that shape the meaning of social exchange processes and, as such, may influence the effects of LMX. In the next section, we introduce the self-concept variables. Then, we develop hypotheses regarding how employee and perceived supervisor self-concept levels influence the relationships of LMX and LMXSC with commitment and, indirectly, performance.

### Levels of the Self-Concept

The self-concept captures the perception that individuals have of themselves (Brewer & Gardner, 1996). It can be approached as an individual difference (i.e., the chronic self) or can be primed by situational cues (i.e., the working self), and it comprises the three distinguishable levels: individual, relational, and collective (Brewer & Gardner, 1996; Johnson, Selenta, & Lord, 2006; Lord & Brown, 2004; Lord, Brown, & Freiberg, 1999; van Knippenberg, van Knippenberg, De Cremer, & Hogg, 2004). At the individual level, people perceive themselves to be unique and independent entities, while at the relational level, the self is defined in terms of dyadic relationships. At the collective level, people define themselves in terms of group membership. This paper focuses on chronic self-conceptions. Thus, we view the self-concept levels as distinguishable, relatively enduring characteristics that exert fairly stable and consistent effects.

The self-concept shapes how information is processed, stored, and retrieved; the goals that individuals set for themselves; and the norms that they follow (Brewer & Gardner, 1996; Lord et al., 1999; van Knippenberg et al., 2004). At the individual level, people focus on what differentiates them from other people; they are driven by a concern for their own welfare, and they seek to achieve personal success (Brewer & Gardner, 1996; Lord & Brown, 2004). At the relational level, people seek to fulfill the expectations of the dyadic partner, attend to his or her needs, and seek to improve their relationship with the partner (Andersen & Chen, 2002), such as the supervisor (Sluss & Ashforth, 2007). At the collective level, behavior is driven by group norms, and collective interest prevails, which leads to a focus on group goals (Johnson, Chang, & Yang, 2010; Lord & Brown, 2004). Thus, the self-concept levels should have independent effects on individuals’ attitudes and behaviors (Brewer & Gardner, 1996). Supporting this view, Johnson et al. (2006) found that only the individual self-concept predicts outcome satisfaction (study 2), only the relational self-concept predicts supervisor satisfaction (study 1), and only the collective self-concept predicts citizenship behavior targeted at the organization (study 2).

### Employee vs. Perceived Supervisor Self-Concept

When employees respond to items about their own self-concept, they provide information on what defines them (Brewer & Gardner, 1996), which can then be used to understand how their self-concept shapes their attitudes and behaviors (e.g., Johnson et al., 2006). In contrast, when employees report their perceptions of their supervisor’s self-concept, they provide information on what they think defines their supervisor (Brewer & Gardner, 1996). In the words of attribution theorists (Jones & Davis, 1965; Kelley & Michela, 1980; Lord & Smith, 1983; Martinko et al., 2007), employees attribute personal characteristics (i.e., self-concept levels) to their supervisor, which, as we argue below, should influence their attitudes and behaviors in their own right.

There are several reasons to think that employees make such attributions. First, it has been suggested that individuals, as naïve psychologists, seek out explanations for their own behavior, other people’s behavior, or specific events to understand, predict, adjust to, or control their environment (Heider, 1958). Thus, making attributions is fundamentally adaptive. Second, individuals are likely to make attributions in contexts that are personally relevant (Martinko et al., 2007). In the work context, employees have a vested interest in explaining their supervisor’s behavior because he or she has a major influence on their work life, for example, through resource allocation (Dépret & Fiske, 1992; Dulebohn et al., 2012; Leikas, Lönnqvist, Verkasalo, & Nissinen, 2013). Finally, individuals are more cognitively predisposed to make internal rather than external attributions when seeking to explain other people’s behavior (i.e., to assume that the cause of other people’s behavior lies within them; Kelley & Michela, 1980; Martinko et al., 2007). This phenomenon, which is referred to as the actor-observer bias (Kelley & Michela, 1980), has been largely documented, including in the context of leader-member relationships (for a discussion, see Martinko et al., 2007). For example, Bernardin (1989) demonstrated that leaders tend to make internal rather than external attributions of members’ unsatisfactory performance. Similarly, employees should be prone to make internal attributions for their supervisor’s behavior. The above arguments suggest that employees view their supervisor’s behavior as a reflection of their self-conception.

We contend that as part of the leader-member relationship, employees observe and interpret the leader’s actions and decisions and associate them with self-concept levels (Dépret & Fiske, 1992; Dulebohn et al., 2012; Leikas et al., 2013). For example, employees may observe the supervisor’s behavior when he or she interacts with different workgroup members and ask “Does my supervisor place a premium on dyadic relationships with other people (i.e., therefore, has a strong relational self-concept)?” Answering this type of question is informative for employees (Jones & Davis, 1965; Kelley & Michela, 1980). It supposes that they view leaders as responsible for their behavior, which leads them to make inferences about the motivations that underlie a specific behavior. For example, if the supervisor is seen as having a strong relational self-concept, employees may infer that the supervisor provides them with additional resources specifically because he or she cares about maintaining a high-quality relationship with them. Thus, making attributions enables employees to give meaning to and predict the leader’s behavior. Making attributions should then influence employees’ attitudes and behaviors in a manner that reflects their understanding of the specific self-concept level that drives the leader’s actions.

### Employee and Perceived Supervisor Relational Self-Concepts as Contingencies

Contrary to LMX, which involves the experience of quality relationships with leaders (Liden & Maslyn, 1998), LMXSC involves a contextualized perception of one’s relationship with the leader. It implies a cognitive processing of the information that pertains to the social comparison process (Vidyarthi et al., 2010). That is, features of one’s relationship with the leader in terms of resources received, trust, and support are gauged against cues related to the parallel relationships of coworkers with the same leader (for a cognitive approach to social judgment, see Mussweiler, 2003). Such a complex cognitive operation is plausibly shaped by the relational level of the self-concept. Indeed, the relational self-concept focuses on the relationship with the dyadic partner (Brewer & Gardner, 1996; Lord et al., 1999).

Employees with a strong relational self-concept should be inclined to value comparative information that is relevant to the dyadic relationship with the supervisor, as conveyed by LMXSC. People with a strong relational self-concept define themselves in terms of dyadic relationships with significant others, strive to fulfill the expectations of their partner, and take to heart meeting the partner’s needs (Andersen & Chen, 2002; Sluss & Ashforth, 2007). For these individuals, self-worth is closely tied to developing a high-quality relationship with the dyadic partner (Johnson et al., 2010). Moreover, the relational self-concept prioritizes the cognitive processing of information related to dyadic relationships (Brewer & Gardner, 1996; Lord et al., 1999). Given that LMXSC speaks to how good the relationship with the supervisor is compared with other employees’ relationship with the supervisor and that the supervisor represents the organization (Eisenberger et al., 2002), we suspect that LMXSC (but not LMX) will exert a stronger effect on organizational commitment at high levels of an employee relational self-concept. Moreover, this moderating effect should extend to work role performance because of organizational commitment’s role as a conduit for reciprocal behavior that targets the organization (Lavelle et al., 2007) and as a driver of performance (Cooper-Hakim & Viswesvaran, 2005; Meyer et al., 2002; Riketta, 2002). The above reasoning leads to the following hypothesis:

- *Hypothesis 2*: The employee relational self-concept will moderate (a) the relationship between LMXSC and organizational commitment and (b) the indirect relationship between LMXSC and work role performance such that these relationships will be stronger (more positive) at high (vs. low) levels of an employee relational self-concept.

As suggested above, employees make attributions about the self-concept level that characterizes their supervisor. They make such internal attributions by interpreting and attending to the supervisor’s behavior, decisions, and actions (Kelley & Michela, 1980; Leikas et al., 2013; Martinko et al., 2007). When the supervisor, for instance, regularly meets with employees to inquire about their needs and offer personalized support, employees are likely to view him or her as valuing dyadic relationships and exerting effort into building mutually beneficial relationships with employees (Chang & Johnson, 2010). Therefore, employees should infer that their supervisor has a strong relational self-concept. We posit that such internal attribution will affect how strongly LMXSC relates to organizational commitment and, in turn, work role performance. Indeed, if the employee perceives that the supervisor holds a strong relational self-concept and, thus, that the supervisor possesses the character of someone who inherently values dyadic relationships (Andersen & Chen, 2002; Brewer & Gardner, 1996), LMXSC will be more meaningful and self-rewarding. A comparatively favorable LMX standing, in this context, would suggest that the supervisor values their dyadic relationship more than the other relationships that he or she has with other employees. Thus, a strong perceived supervisor relational self-concept gives substance to LMXSC. LMXSC should, as a result, lead to a stronger feeling of obligation to reciprocate through organizational commitment and, indirectly, through work performance when the perceived supervisor relational self-concept is high. This reasoning is summarized in the following hypothesis:

- *Hypothesis 3*: The perceived supervisor relational self-concept will moderate (a) the relationship between LMXSC and organizational commitment and (b) the indirect relationship between LMXSC and work role performance such that these relationships will be stronger (more positive) at high (vs. low) levels of perceived supervisor relational self-concept.

### Perceived Supervisor Collective Self-Concept as a Contingency

The collective self-concept may be particularly relevant when it is attributed to the supervisor and likely has implications for LMX and LMXSC. The collective self-concept involves self-definition based on one’s membership in groups (Brewer & Gardner, 1996; Johnson et al., 2010). Because it is self-defining, the belongingness to, and success of, the group fosters self-worth among people with a high collective self-concept. Moreover, a collective self-concept sensitizes individuals to the norms, goals, and values of the group to which they belong so that they naturally defend it in the face of adversity (Ellemers, De Gilder, & Haslam, 2004). Such self-concept level also makes the characteristics of the group salient (Hogg & Terry, 2000). Thus, when employees view the supervisor’s actions and decisions as being driven by a concern for the welfare of the organization and its members (Brewer & Gardner, 1996), such as when he or she takes the lead of demanding projects that promote the organization’s goals and values, they should infer that the supervisor has a strong collective self-concept.

The perceived supervisor collective self-concept should indicate to employees that the relationship with the leader strongly represents the relationship with the organization itself (Eisenberger et al., 2010). When supervisors put organizational interests first and defend them, they should be seen not only as formal representatives of the organization but also as authentic and pivotal in-group members (Ellemers et al., 2004). Thus, when employees perceive that they have a high-quality relationship with the leader (i.e., LMX) or a comparatively high-quality relationship with the leader (i.e., LMXSC) and see the leader as having a strong collective self-concept, they should feel more strongly connected to the organization. The leader-member relationship should more closely symbolize the relationship with the organization in such cases. Accordingly, when experiencing high LMX or LMXSC, these employees should feel more obligated to reciprocate. Therefore, LMX and LMXSC should be more strongly related to organizational commitment and indirectly to work role performance when the supervisor is perceived to hold a strong collective self-concept. In contrast, the employee collective self-concept is not expected to have a similar moderating effect. The supervisor is perceived by employees to represent the organization, and the effect of the perceived supervisor collective self-concept should, as argued above, operate through attribution processes. The perceived supervisor collective self-concept reflects employees’ attribution of characteristics to the supervisor, while the employee self-concept reflects self-perception (Brewer & Gardner, 1996; Jones & Davis, 1965; Kelley & Michela, 1980; Lord & Smith, 1983; Martinko et al., 2007). Thus, the following hypotheses are proposed:

- *Hypothesis 4*: The perceived supervisor collective self-concept will moderate (a) the relationship between LMX and organizational commitment and (b) the indirect relationship between LMX and work role performance such that these relationships will be stronger (more positive) at high (vs. low) levels of perceived supervisor collective self-concept.
- *Hypothesis 5*: The perceived supervisor collective self-concept will moderate (a) the relationship between LMXSC and organizational commitment and (b) the indirect relationship between LMXSC and work role performance such that these relationships will be stronger (more positive) at high (vs. low) levels of perceived supervisor collective self-concept.

## The Present Study

**Fig. 1:**
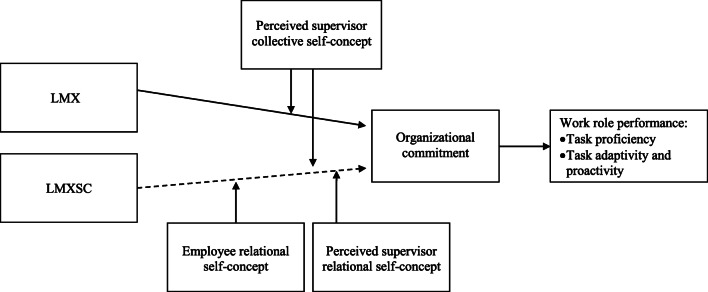
Summary of the research model

In the present study, we postulate that LMX will predict work role performance through increased organizational commitment. We also expect that among the self-concept variables, the relational and collective levels will act as significant moderators of the relationships among LMX, LMXSC, and work outcomes. We use matched data from employees and supervisors to examine these relationships, which are depicted in Fig. 1.

## Method

### Sample and Procedure

We obtained consent from the managers of customer service departments from organizations located in Canada to conduct a study on work attitudes among their staff. These organizations operated in the fields of electronic equipment (*n* = 2), insurance (*n* = 1), telecommunications (*n* = 2), and food and consumer goods retailing (*n* = 2). The agreement was that employees would receive a questionnaire that included, among others, measures of LMX, LMXSC, the employee and perceived supervisor individual, relational, and collective levels of the self-concept, and organizational commitment. An accompanying letter described the purpose of the study and ensured the respondents that participation was voluntary and the responses were confidential. The respondents received a $5 gift card as a compensation for their time. In addition, the department managers (*n* = 7) separately completed a questionnaire that contained the work role performance scale items for each employee. As managers were to rate the performance of multiple employees, we gave them 3 weeks to do this and compensated them via a $30 gift card. The employees’ and managers’ questionnaires were coded so that the responses could be matched for purposes of analysis.

Among the 370 questionnaires distributed to employees and department managers, matched responses were obtained for 250 employees (for an overall 67.6% response rate; the rates effectively ranged from 52.2 to 82.0% across departments). In this final sample, 66.8% of the employees were male, with an average age of 33.18 years (*SD* = 5.32), an average organizational tenure of 3.14 years (*SD* = 3.33), and an average tenure with the supervisor of 2.08 years (*SD* = 1.80). In terms of education, 93.1% of the employees had at least a 2-year college level, while the remaining 6.9% had a high school level. Most employees (83.33%) worked full-time. Among managers, four were women, average age was 45.43 years (*SD* = 4.54; range = 41–52), and average organizational tenure was 10.71 years (*SD* = 3.45; range = 7–16).

### Measures

A 5-point Likert-type scale (1 = *strongly disagree*, 5 = *strongly agree*) was used for all substantive items. As the respondents were French-speaking, we used a translation-back-translation procedure to translate from English to French the items that were not available in French.

#### LMX

To assess LMX, we used Liden and Maslyn’s (1998) LMX-MDM 12-item scale, which contains four subscales that capture affect, loyalty, contribution, and professional respect. As these subscales are generally highly correlated with one another, it is common to use a single score of LMX by averaging across the items of the scale (e.g., Erdogan & Enders, 2007). A sample item is “I am impressed with my manager’s knowledge of his/her job” (respect dimension). The LMX scale had an overall alpha coefficient of .92 in this study.

#### LMXSC

We used a 6-item scale initially developed by Erdogan (2002) and reported in Vidyarthi et al. (2010) to assess LMXSC. A sample item is “My manager is more loyal to me compared to my coworkers.” The alpha coefficient for this scale was .90 in this study.

#### Employee and Perceived Supervisor Self-Concepts

We used the Levels of the Self-Concept Scale (LSCS) developed by Selenta and Lord (2005), which was further validated since then (e.g., Johnson et al., 2006), to capture the self-concept levels. The scales of the employee individual (e.g., “I often compete with my friends”), relational (e.g., “I value friends who are caring, empathic individuals”), and collective (e.g., “When I become involved in a group project, I do my best to ensure its success”) levels of the self-concept included five items each. All scales exhibited good reliability in this study (*α*s = .80, .85, and .78, respectively). To measure the perceived supervisor self-concept at the different levels, we adapted the items in each scale so that they reflected employees’ perceptions. For example, the item “I often compete with my friends” was reworded as “My supervisor likes to compete with others.” The alpha coefficients of the three levels of perceived supervisor self-concept scales, which included five items each, were reasonably good, namely, .81, .78, and .74 for the individual, relational, and collective levels of the self-concept, respectively.

#### Organizational Commitment

A French version (Bentein, Vandenberg, Vandenberghe, & Stinglhamber, 2005) of Meyer, Allen, and Smith’s (1993) 6-item scale was used to measure organizational commitment. A sample item is “I feel like part of the family at this organization.” The alpha coefficient for this scale was .93 in this study.

#### Work Role Performance

Work performance has been conceptualized in various ways over the years (Carpini, Parker, & Griffin, 2017). To capture performance in this study, we focused on the dimensions of individual work role performance outlined by Griffin et al. (2007). These authors distinguish individual task proficiency (i.e., the extent to which employees meet formal job requirements) from individual task adaptivity (i.e., the extent to which employees adapt to changes in work roles) and individual task proactivity (i.e., the extent to which employees take self-directed action to anticipate or initiate change in work roles). We measured these performance dimensions through supervisor ratings by using the three 3-item scales provided by Griffin et al. (2007). Sample items are “This employee completed his/her core tasks well using the standard procedures” (task proficiency), “This employee adapted well to changes in core tasks” (task adaptivity), and “This employee made changes to the way his/her core tasks are done” (task proactivity).

Consistent with Griffin et al.’s (2007) conceptualization, we initially approached the three performance dimensions as distinct outcomes. However, recent research has indicated that task proficiency on the one hand and task adaptivity and proactivity on the other hand constitute two instead of three separate factors (Vandenberghe, Mignonac, & Manville, 2015) that relate to unique antecedents (Griffin, Parker, & Mason, 2010). Research also suggested that task proficiency might be more relevant in a low-uncertainty context, while task adaptivity and proactivity might be more relevant in a high-uncertainty context (Carpini et al., 2017; Griffin et al., 2007). Thus, we examined the structure of the nine performance items by using confirmatory factor analysis (CFA) through LISREL 8.80 (Jöreskog, Sörbom, Du Toit, & Du Toit, 2001) and the maximum likelihood method of estimation. A three-factor model yielded a good fit to the data, *χ*^2^ (22) = 60.16, *p* < .001, root mean square error of approximation (RMSEA) = .086, comparative fit index (CFI) = .99, Tucker-Lewis index (TLI) = .99, and standardized root mean square residual (SRMR) = .03. Although this model outperformed a two-factor model in which task adaptivity and proactivity were merged (Δ*χ*^2^ [2] = 26.10, *p* < .001), the correlation between these two factors in the three-factor solution was extremely high (*r* = .99), which suggested that they formed a single factor. We thus retained the two-factor solution. The 3-item scale of task proficiency had an alpha coefficient of .95, while the 6-item scale of task adaptivity and proactivity had an alpha coefficient of .89.

#### Control Variables

Age, gender, organizational tenure, and tenure with the supervisor were controlled for as these variables were found to be related to LMX and LMXSC (Vidyarthi et al., 2010), organizational commitment (Meyer et al., 2002), performance (Griffin et al., 2007), and the quality of employee-supervisor relationships (Harris, Kacmar, & Carlson, 2006).

## Results

### Confirmatory Factor Analyses

We first examined the discriminant validity of our measurement model by using CFA through LISREL 8.80 (Jöreskog et al., 2001) and the maximum likelihood method of estimation. Due to the complexity of our model (i.e., 63 items), we created three item parcels for all constructs measured by more than three items. We used the high-to-low loadings procedure outlined in Little, Cunningham, Shahar, and Widaman (2002) to assign items to parcels. We compared the fit of the theoretical model to a number of nested, more parsimonious models by using Δ*χ*^2^ tests. The results are presented in Table 1. The hypothesized 11-factor model yielded a good fit, *χ*^2^ (440) = 644.16, *p* < .001, RMSEA = .04, CFI = .96, TLI = .95, SRMR = .05. This model outperformed a series of 10-factor models that merged the factors on a two-by-two basis (Δ*χ*^2^ = 133.09 to 477.75, Δ*df* = 10, *p* < .001), 9-factor models that combined either employee (Δ*χ*^2^ [19] = 326.89, *p* < .001) or perceived supervisor (Δ*χ*^2^ [19] = 359.87, *p* < .001) self-concepts, a 3-factor model that merged employee-reported measures (Δ*χ*^2^ [52] = 2177.48, *p* < .001), a 2-factor model (employee- vs. supervisor-reported measures) (Δ*χ*^2^ [54] = 2649.71, *p* < .001), and a one-factor model (Δ*χ*^2^ [55] = 3551.65, *p* < .001). This attests to the discriminant validity of our variables.

**Table 1:** Confirmatory factor analysis of the measurement models: fit indices

Model	*χ*^2^	*df*	Δ*χ*^2^	Δ*df*	RMSEA	CFI	TLI	SRMR
1. Hypothesized 11-factor model	644.16***	440	–	–	.04	.96	.95	.05
2. Ten-factor models								
Combining LMX and LMXSC	1077.65***	450	433.49***	10	.08	.88	.86	.07
Combining task proficiency and task adaptivity/proactivity	1121.91***	450	477.75***	10	.08	.87	.85	.06
Combining employee and perceived supervisor individual self-concept	819.18***	450	175.02***	10	.06	.93	.92	.06
Combining employee and perceived supervisor relational self-concept	1055.66***	450	411.50***	10	.07	.88	.86	.08
Combining employee and perceived supervisor collective self-concept	777.25***	450	133.09***	10	.05	.94	.93	.06
3. Nine-factor models								
Combining employee’s self-concepts	971.05***	459	326.89***	19	.07	.90	.89	.08
Combining perceived supervisor’s self-concepts	1004.03***	459	359.87***	19	.07	.89	.88	.08
4. Three-factor model								
Combining employee-reported measures	2821.64***	492	2177.48***	52	.14	.54	.51	.13
5. Two-factor model								
Employee-reported measures vs. supervisor-reported measures	3293.87***	494	2649.71***	54	.15	.45	.41	.14
6. One-factor model	4195.81***	495	3551.65***	55	.17	.28	.23	.17

*N* = 250

*LMX* leader-member exchange, *LMXSC* leader-member exchange social comparison, *RMSEA* root mean square error of approximation, *CFI* comparative fit index, *TLI* Tucker-Lewis index, *SRMR* standardized root mean square residual

***p < .001

### Descriptive Statistics and Intercorrelations

The descriptive statistics, reliabilities, and intercorrelations for our variables are presented in Table 2. All variables displayed good internal consistency (*α*s ≥ .74). LMX and LMXSC positively correlated with employee individual self-concept (*r* = .17 vs. .27, *p*s < .01), the perceived supervisor individual (*r* = .14, *p* < .05, vs. .26, *p* < .01) and collective (*r*s = .18, *p*s < .01) self-concepts, organizational commitment (*r* = .43 vs. .25, *p*s < .01), task proficiency (*r*s = .15, *p*s < .05), and task adaptivity and proactivity (*r* = .30 vs. .20, *p*s < .01). Commitment was positively related to task proficiency (*r* = .30, *p* < .01) and task adaptivity and proactivity (*r* = .31, *p* < .01).

**Table 2:** Descriptive statistics and intercorrelations

Variable	*M*	*SD*	1	2	3	4	5	6	7	8	9	10	11	12	13	14	15
1. Age	33.18	5.32	–														
2. Gender	1.67	0.47	.15*	–													
3. Organizational tenure	3.14	3.33	.23**	− .03	–												
4. Tenure with supervisor	2.08	1.80	.19**	− .17**	.77**	–											
5. LMX	3.45	0.79	.00	− .08	.15*	.09	(.92)										
6. LMXSC	2.93	0.99	− .03	− .13*	.13*	.14*	.42**	(.90)									
7. Employee individual self-concept	3.37	0.85	.05	− .02	.00	.02	.17**	.27**	(.80)								
8. Employee relational self-concept	4.11	0.76	.04	.00	− .03	− .06	.02	− .10	.34**	(.85)							
9. Employee collective self-concept	3.99	0.70	.12	− .02	.05	.06	.27**	.08	.29**	.47**	(.78)						
10. Perceived supervisor individual self-concept	3.14	0.95	.06	− .03	.14*	.14*	.14*	.26**	.35**	.04	.04	(.81)					
11. Perceived supervisor relational self-concept	3.67	0.81	− .06	− .07	.03	.03	.49**	.10	.14*	.10	.22**	.11	(.78)				
12. Perceived supervisor collective self-concept	3.97	0.78	− .03	− .07	− .02	− .05	.18**	.18**	.14*	.17**	.25**	.13*	.25**	(.74)			
13. Organizational commitment	3.59	1.03	− .05	− .04	.11	.06	.43**	.25**	.03	− .05	.17**	.13*	.43**	.32**	(.93)		
14. Task proficiency	4.07	0.98	.02	− .05	.49**	.44**	.15*	.15*	.00	− .04	.17**	.11	.07	.07	.30**	(.95)	
15. Task adaptivity and proactivity	2.87	1.10	− .05	− .11	.46**	.43**	.30**	.20**	.01	− .02	.16*	.15*	.22**	.12	.31**	.65**	(.89)

*N*s = 232–239. For gender, 1 = female, 2 = male. Alpha coefficients are reported in parentheses along the diagonal

*LMX* leader-member exchange, *LMXSC* leader-member exchange social comparison

*p < .05, **p < .01

### Hypothesis Testing

As employees belonged to various departments and as managers rated the performance of multiple employees, there might be a nonindependence issue in the data. To account for the possibility of manager/department effects, we used hierarchical linear modeling (HLM; Raudenbush & Bryk, 2002) to test all hypotheses. We estimated two-level models with employees being nested within departments and used the restricted maximum likelihood (REML) method of estimation, which provides robust estimates when the number of units is small (*n* = 7) (Dedrick et al., 2009; McNeish & Stapleton, 2016) to compare the fixed-effects models (Snijders & Bosker, 2012). We first conducted unconditional HLM models (i.e., with no predictors) for organizational commitment, task proficiency, and task adaptivity and proactivity. The variance component of these intercept-only models was significant for commitment (*χ*^2^ [6] = 16.14, *p* < .05; *R*^2^ = .05) and task proficiency (Δ*χ*^2^ [6] = 25.72, *p* < .001; *R*^2^ = .09) but nonsignificant, yet close to significance, for task adaptivity and proactivity (Δ*χ*^2^ [6] = 12.48, *p* = .052; *R*^2^ = .04). Following LeBreton and Senter (2008), a value (i.e., variance explained) of .01 should be considered to be a small effect, a value of .10 should be considered to be a medium effect, and a value of .25 should be considered to be a large effect. The values reported above fall in the range of small to medium effects, which justifies the use of multilevel modeling. As our predictors were all at level 1, the residual variance parameters for the level 1 predictors were set to zero (Snijders & Bosker, 2012). Thus, although our model contained no predictors at level 2, such a procedure allowed controlling for department-level dependencies in the data. As recommended by Hofmann and Gavin (1998), the variables were grand-mean centered, which helped reduce multicollinearity when testing the effect of the interactions among the level 1 predictors.

Hypothesis 1 predicted that organizational commitment would mediate a positive relationship between LMX and performance. Table 3 (model 1) displays the results of the HLM analysis for organizational commitment, including the control variables, all self-concept levels, LMX, and LMXSC as predictors. As seen, LMX was positively related to organizational commitment (*γ* = .28, *p* < .01). Next, Table 4 (model 1) reports the results of the HLM analysis for task proficiency in which organizational commitment was introduced as a predictor in addition to the same set of predictors as in Table 3 (model 1). In this analysis, organizational commitment was positively related to task proficiency (*γ* = .22, *p* < .001), while the effects of LMX (*γ* = − .05, *ns*) and LMXSC (*γ* = .03, *ns*) were nonsignificant. Similarly, Table 5 (model 1) reports the results of the HLM analysis for task adaptivity and proactivity by using the same set of variables as predictors. As seen, organizational commitment was positively related to task adaptivity and proactivity (*γ* = .17, *p* < .05), while the effects of LMX (*γ* = .20, *ns*) and LMXSC (*γ* = .01, *ns*) were nonsignificant. We then used Hayes’s (2013) PROCESS macro for SPSS and 5000 bootstrapped resamples to test the indirect relationships between LMX and performance dimensions through commitment and their associated confidence intervals (CIs). The indirect effect was found to be significant in predicting both task proficiency (.07; CI [.018, .152], *p* < .05) and task adaptivity and proactivity (.05; CI [.006, .124], *p* < .05).^2^ Hypothesis 1 is thus supported.^3^^,^^4^

**Table 3:** Hierarchical linear modeling results for organizational commitment

Variable	Model 1	Model 2	Model 3	Model 4
Intercept *γ*_00_	3.60***	3.60***	3.58***	3.60***
Employee age *γ*_10_	− 0.00	− 0.00	0.00	− 0.01
Employee gender *γ*_20_	− 0.00	− 0.04	− 0.02	0.01
Employee organizational tenure *γ*_30_	0.02	0.02	0.02	0.02
Employee tenure with supervisor *γ*_40_	0.01	0.01	0.02	0.02
LMX *γ*_50_	0.28**	0.34***	0.33***	0.30**
LMXSC *γ*_60_	0.06	0.07	0.04	0.04
Employee individual self-concept *γ*_70_	− 0.13	− 0.11	− 0.13	− 0.12
Employee relational self-concept *γ*_80_	− 0.18*	− 0.18*	− 0.23**	− 0.19*
Employee collective self-concept *γ*_90_	0.19	0.17	0.20*	0.22*
Perceived supervisor individual self-concept *γ*_100_	0.04	− 0.02	0.02	0.02
Perceived supervisor relational self-concept *γ*_110_	0.35***	0.36***	0.30***	0.34***
Perceived supervisor collective self-concept *γ*_120_	0.21*	0.24**	0.22**	0.22*
LMX × employee individual self-concept *γ*_130_		− 0.10		
LMX × perceived supervisor individual self-concept *γ*_140_		0.20*		
LMXSC × employee individual self-concept *γ*_150_		0.17*		
LMXSC × perceived supervisor individual self-concept *γ*_160_		− 0.22***		
LMX × employee relational self-concept *γ*_170_			0.02	
LMX × perceived supervisor relational self-concept *γ*_180_			0.07	
LMXSC × employee relational self-concept *γ*_190_			0.17*	
LMXSC × perceived supervisor relational self-concept *γ*_200_			0.16*	
LMX × employee collective self-concept *γ*_210_				0.06
LMX × perceived supervisor collective self-concept *γ*_220_				− 0.22*
LMXSC × employee collective self-concept *γ*_230_				− 0.01
LMXSC × perceived supervisor collective self-concept *γ*_240_				0.18*
Δ*R*^2^	0.30	0.04	0.03	0.01
Deviance	615.15	614.22	613.47	620.22

*N* (level 1) = 233; *N* (level 2) = 7. For gender, 1 = female, 2 = male. Unstandardized coefficients are reported. *R*^2^ values are computed as the proportional reduction in the levels 1 and 2 error variance due to the predictors (Snijders & Bosker, 2012, p. 306)

*LMX* leader-member exchange, *LMXSC* leader-member exchange social comparison

*p < .05, **p < .01, ***p < .001

**Table 4:** Hierarchical linear modeling results for task proficiency

Variable	Model 1	Model 2	Model 3	Model 4
Intercept *γ*_00_	3.30***	4.10***	4.13***	4.11***
Employee age *γ*_10_	− 0.02	− 0.02	− 0.02	− 0.02
Employee gender *γ*_20_	0.04	0.01	0.05	0.04
Employee organizational tenure *γ*_30_	0.09***	0.09***	0.09***	0.09***
Employee tenure with supervisor *γ*_40_	0.11*	0.10*	0.10*	0.11*
LMX *γ*_50_	− 0.05	0.01	− 0.09	− 0.02
LMXSC *γ*_60_	0.03	0.02	0.03	0.03
Employee individual self-concept *γ*_70_	− 0.05	− 0.05	− 0.06	− 0.04
Employee relational self-concept *γ*_80_	− 0.09	− 0.09	− 0.06	− 0.09
Employee collective self-concept *γ*_90_	0.26**	0.24**	0.30**	0.23*
Perceived supervisor individual self-concept *γ*_100_	0.02	0.02	0.02	0.03
Perceived supervisor relational self-concept *γ*_110_	− 0.07	− 0.05	− 0.07	− 0.08
Perceived supervisor collective self-concept *γ*_120_	0.00	0.01	− 0.01	0.02
LMX × employee individual self-concept *γ*_130_		− 0.08		
LMX × perceived supervisor individual self-concept *γ*_140_		0.16*		
LMXSC × employee individual self-concept *γ*_150_		0.02		
LMXSC × perceived supervisor individual self-concept γ_160_		− 0.06		
LMX × employee relational self-concept *γ*_170_			0.13	
LMX × perceived supervisor relational self-concept *γ*_180_			− 0.10	
LMXSC × employee relational self-concept *γ*_190_			− 0.12	
LMXSC × perceived supervisor relational self-concept *γ*_200_			0.02	
LMX × employee collective self-concept *γ*_210_				− 0.08
LMX × perceived supervisor collective self-concept *γ*_220_				0.08
LMXSC × employee collective self-concept *γ*_230_				− 0.01
LMXSC × perceived supervisor collective self-concept *γ*_240_				− 0.01
Organizational commitment *γ*_250_	0.22***	0.20**	0.24***	0.23***
Δ*R*^2^	0.32	0.01	0.00	0.00
Deviance	598.66	608.74	608.00	609.28

*N* (level 1) = 233; *N* (level 2) = 7. For gender, 1 = female, 2 = male. Unstandardized coefficients are reported. *R*^2^ values are computed as the proportional reduction in the levels 1 and 2 error variance due to the predictors (Snijders & Bosker, 2012, p. 306)

*LMX* leader-member exchange, *LMXSC* leader-member exchange social comparison

*p < .05, **p < .01, ***p < .001

**Table 5:** Hierarchical linear modeling results for task adaptivity and proactivity

Variable	Model 1	Model 2	Model 3	Model 4
Intercept *γ*_00_	2.29***	2.92***	2.93***	2.92***
Employee age *γ*_10_	− 0.04**	− 0.04**	− 0.04**	− 0.04**
Employee gender *γ*_20_	− 0.01	− 0.02	0.00	− 0.01
Employee organizational tenure *γ*_30_	0.10***	0.10***	0.10***	0.10***
Employee tenure with supervisor *γ*_40_	0.12*	0.11*	0.11*	0.12*
LMX *γ*_50_	0.20	0.22*	0.16	0.20
LMXSC *γ*_60_	0.01	− 0.00	0.02	− 0.01
Employee individual self-concept *γ*_70_	− 0.13	− 0.14	− 0.14	− 0.13
Employee relational self-concept *γ*_80_	− 0.00	0.01	0.02	− 0.02
Employee collective self-concept *γ*_90_	0.16	0.15	0.19	0.18
Perceived supervisor individual self-concept *γ*_100_	0.08	0.10	0.08	0.08
Perceived supervisor relational self-concept *γ*_110_	0.05	0.06	0.05	0.07
Perceived supervisor collective self-concept *γ*_120_	0.01	− 0.00	0.00	0.01
LMX × employee individual self-concept *γ*_130_		− 0.03		
LMX × perceived supervisor individual self-concept *γ*_140_		0.06		
LMXSC × employee individual self-concept *γ*_150_		− 0.08		
LMXSC × perceived supervisor individual self-concept *γ*_160_		0.02		
LMX × employee relational self-concept *γ*_170_			0.09	
LMX × perceived supervisor relational self-concept *γ*_180_			− 0.08	
LMXSC × employee relational self-concept *γ*_190_			− 0.08	
LMXSC × perceived supervisor relational self-concept *γ*_200_			0.01	
LMX × employee collective self-concept *γ*_210_				− 0.03
LMX × perceived supervisor collective self-concept *γ*_220_				− 0.08
LMXSC × employee collective self-concept *γ*_230_				0.06
LMXSC × perceived supervisor collective self-concept *γ*_240_				0.01
Organizational commitment *γ*_250_	0.17*	0.18*	0.19*	0.17*
Δ*R*^2^	0.33	0.00	0.00	0.00
Deviance	649.51	660.50	659.80	659.28

*N* (level 1) = 233; *N* (level 2) = 7. For gender, 1 = female, 2 = male. Unstandardized coefficients are reported. *R*^2^ values are computed as the proportional reduction in the levels 1 and 2 error variance due to the predictors (Snijders & Bosker, 2012, p. 306)

*LMX* leader-member exchange, *LMXSC* leader-member exchange social comparison

*p < .05, **p < .01, ***p < .001

Our next hypotheses involved two-way interaction effects. As both LMX and LMXSC were included in these interactions and we had three levels of the self-concept for employees and their supervisors, there were 12 potential interactions to be tested. To maintain sufficient power in testing the moderation effects (Aguinis & Gottfredson, 2010), we tested the interactions for each level of the self-concept separately while still controlling for the main effect of all employee and perceived supervisor self-concept levels, which is common practice (e.g., Johnson et al., 2006). Thus, there were three moderated HLM models for organizational commitment (Table 3, models 2–4), task proficiency (Table 4, models 2–4), and task adaptivity and proactivity (Table 5, models 2–4).

**Fig. 2:**
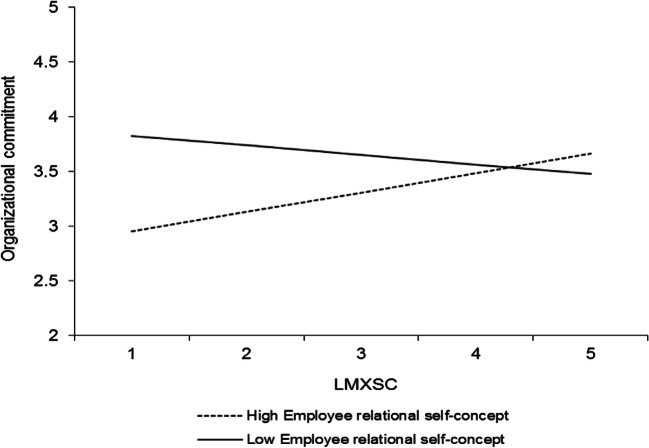
Interaction between LMXSC and employee relational self-concept in predicting organizational commitment. Slopes are reported at 1 *SD* above and below the mean of the moderator

Hypothesis 2 predicted that LMXSC would interact with the employee relational self-concept such that (a) its relationship with organizational commitment and (b) its indirect relationship with performance would be stronger at higher levels of the employee relational self-concept. To test this hypothesis, we ran a HLM analysis that predicted organizational commitment in which the control variables, all self-concept levels, LMX, and LMXSC were included as predictors along with the interactions between LMX and LMXSC and the employee and perceived supervisor relational self-concepts. The results are presented in Table 3 (model 3). As seen, LMXSC significantly interacted with the employee relational self-concept (*γ* = .17, *p* < .05). To illustrate the form of this interaction, we plotted the regression line for organizational commitment on LMXSC at 1 *SD* below and 1 *SD* above the mean of the employee relational self-concept (cf. Aiken & West, 1991) (Fig. 2). Simple slopes analyses using Preacher, Curran, and Bauer’s (2006) approach showed that LMXSC was unrelated to organizational commitment at low levels (− 1 *SD*) of the employee relational self-concept (*γ* = − .13, *t* = − 1.13, *ns*) but positively related to organizational commitment at high levels (+ 1 *SD*) of the employee relational self-concept (*γ* = .22, *t* = 2.25, *p* < .05). Hypothesis 2a is therefore supported.

To examine whether the employee relational self-concept also moderated the indirect relationship between LMXSC and performance through commitment (i.e., moderated mediation), we used Hayes’s (2013) PROCESS macro to estimate the conditional indirect effects based on 5000 bootstrapped resamples.^5^ The moderated mediation was nonsignificant for both task proficiency (.04; CI [− .001, .121], *ns*) and task adaptivity and proactivity (.03; CI [− .001, .093], *ns*). Thus, although the employee relational self-concept moderated the LMXSC-commitment relationship in a manner consistent with our expectations, this effect did not extend to the indirect relationships between LMXSC and performance dimensions. Thus, hypothesis 2b is not supported.

**Fig. 3:**
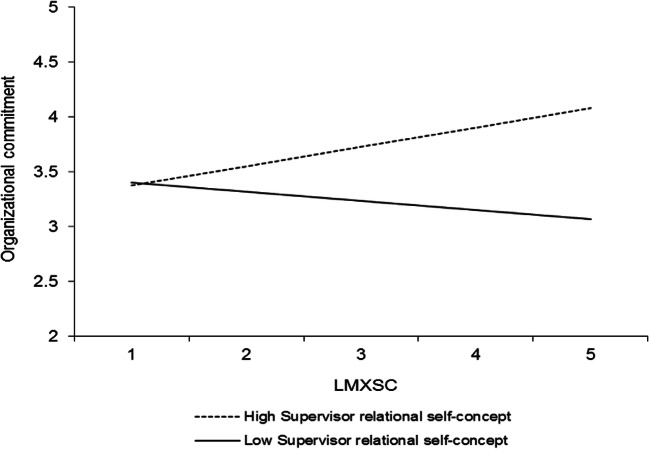
Interaction between LMXSC and perceived supervisor relational self-concept in predicting organizational commitment. Slopes are reported at 1 *SD* above and below the mean of the moderator

Hypothesis 3 proposed that LMXSC would interact with the perceived supervisor relational self-concept such that (a) its relationship with organizational commitment and (b) its indirect relationship with performance would be stronger at higher levels of this moderator. Using the same HLM model as for hypothesis 2 (Table 3, model 3), LMXSC was found to interact significantly with the perceived supervisor relational self-concept in predicting organizational commitment (*γ* = .16, *p* < .05). This interaction is graphed in Fig. 3. Simple slopes analyses (Preacher et al., 2006) revealed that LMXSC was unrelated to organizational commitment at low levels (− 1 *SD*) of the perceived supervisor relational self-concept (*γ* = − .11, *t* = − 1.16, *ns*) but positively related to organizational commitment at high levels (+ 1 *SD*) of the perceived supervisor relational self-concept (*γ* = .21, *t* = 2.15, *p* < .05). Therefore, hypothesis 3a is supported.

We then conducted moderated mediation analyses using Hayes’s (2013) PROCESS macro and 5000 bootstrapped resamples to examine whether the perceived supervisor relational self-concept also moderated the indirect effect of LMXSC on the performance outcomes. The moderated mediation was nonsignificant for both task proficiency (.04; CI [− .009, .097], *ns*) and task adaptivity and proactivity (.03; CI [− .008, .088], *ns*). Thus, the perceived supervisor relational self-concept moderated the LMXSC-commitment relationship in a manner consistent with our expectations, but this effect did not extend to the indirect relationship between LMXSC and performance. Hypothesis 3b is thus not supported.

**Fig. 4:**
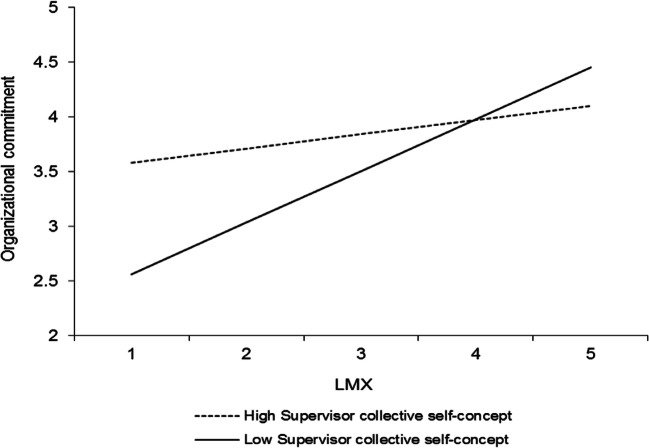
Interaction between LMX and perceived supervisor collective self-concept in predicting organizational commitment. Slopes are reported at 1 *SD* above and below the mean of the moderator

Hypothesis 4 predicted that LMX would interact with the perceived supervisor collective self-concept such that (a) its relationship with organizational commitment and (b) its indirect relationship with work role performance would be stronger at higher levels of the perceived supervisor collective self-concept. Table 3 (model 4) displays the results of a HLM analysis that predicts organizational commitment in which the control variables, self-concept levels, LMX, and LMXSC were included as predictors along with the interactions between LMX and LMXSC and the employee and perceived supervisor collective self-concepts. As seen, LMX interacted with the perceived supervisor collective self-concept to predict organizational commitment (*γ* = − .22, *p* < .05). The interaction is graphed in Fig. 4. Simple slopes analyses (Preacher et al., 2006) revealed that LMX was unrelated to organizational commitment at high levels (+ 1 *SD*) of the perceived supervisor collective self-concept (*γ* = .08, *t* = 0.56, *ns*) but positively related to organizational commitment at low levels (− 1 *SD*) of the perceived supervisor collective self-concept (*γ* = .52, *t* = 3.91, *p* < .0001), which reflects a pattern that contradicts hypothesis 4a. Thus, it is not supported.

Using Hayes’s (2013) PROCESS macro and 5000 bootstrapped resamples, we also found the perceived supervisor collective self-concept to significantly moderate the indirect effect of LMX on task proficiency (− .05; CI [− .143, − .006], *p* < .05) and task adaptivity and proactivity (− .04; CI [− .123, − .001], *p* < .05). The indirect effect of LMX on task proficiency and task adaptivity and proactivity was nonsignificant at high levels of the perceived supervisor collective self-concept (.03; CI [− .028, .110], *ns*, and .02; CI [− .017, .089], *ns*, respectively) but significantly positive at low levels of the perceived supervisor collective self-concept (.11; CI [.035, .226], *p* < .05; and .08; CI [.009, .186], *p* < .05, respectively). The pattern of this effect contradicts hypothesis 4b. Therefore, it is not supported.

**Fig. 5:**
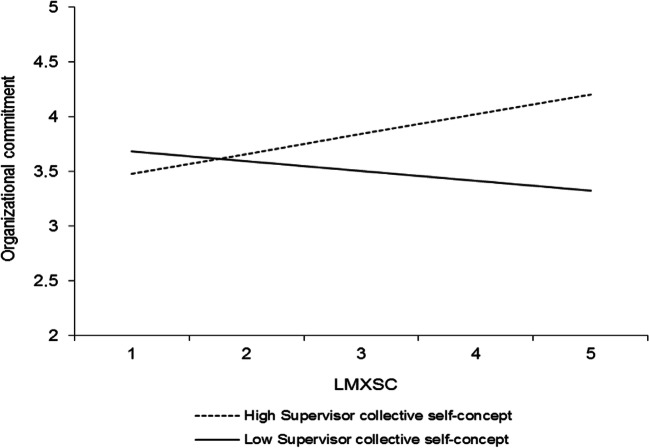
Interaction between LMXSC and perceived supervisor collective self-concept in predicting organizational commitment. Slopes are reported at 1 *SD* above and below the mean of the moderator

Hypothesis 5 predicted that LMXSC would interact with the perceived supervisor collective self-concept such that (a) its relationship with organizational commitment and (b) its indirect relationship with performance would be stronger at higher levels of this moderator. Table 3 (model 4) displays the results of the moderated HLM analysis that predicts organizational commitment. As seen, LMXSC interacted with the perceived supervisor collective self-concept to predict commitment (*γ* = .18, *p* < .05). The interaction is illustrated in Fig. 5. Simple slopes analyses (Preacher et al., 2006) indicated that LMXSC was unrelated to organizational commitment at low levels (− 1 *SD*) of the perceived supervisor collective self-concept (*γ* = − .13, *t* = − 1.21, *ns*) but positively related to organizational commitment at high levels (+ 1 *SD*) of the perceived supervisor collective self-concept (*γ* = .22, *t* = 2.08, *p* < .05). Hypothesis 5a is thus supported. We used Hayes’s (2013) PROCESS macro and 5000 bootstrapped resamples to examine if the perceived supervisor collective self-concept moderated the indirect effect of LMXSC on task proficiency, and task adaptivity and proactivity. Both effects were found to be nonsignificant (.04; CI [− .001, .109], *ns*, and .03; CI [− .002, .083], *ns*, respectively). Therefore, hypothesis 5b is not supported.^6^

### Ancillary Results

**Fig. 6:**
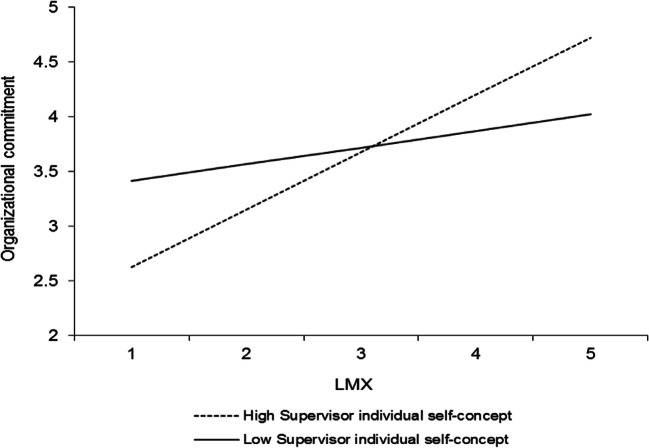
Interaction between LMX and perceived supervisor individual self-concept in predicting organizational commitment. Slopes are reported at 1 *SD* above and below the mean of the moderator

**Fig. 7:**
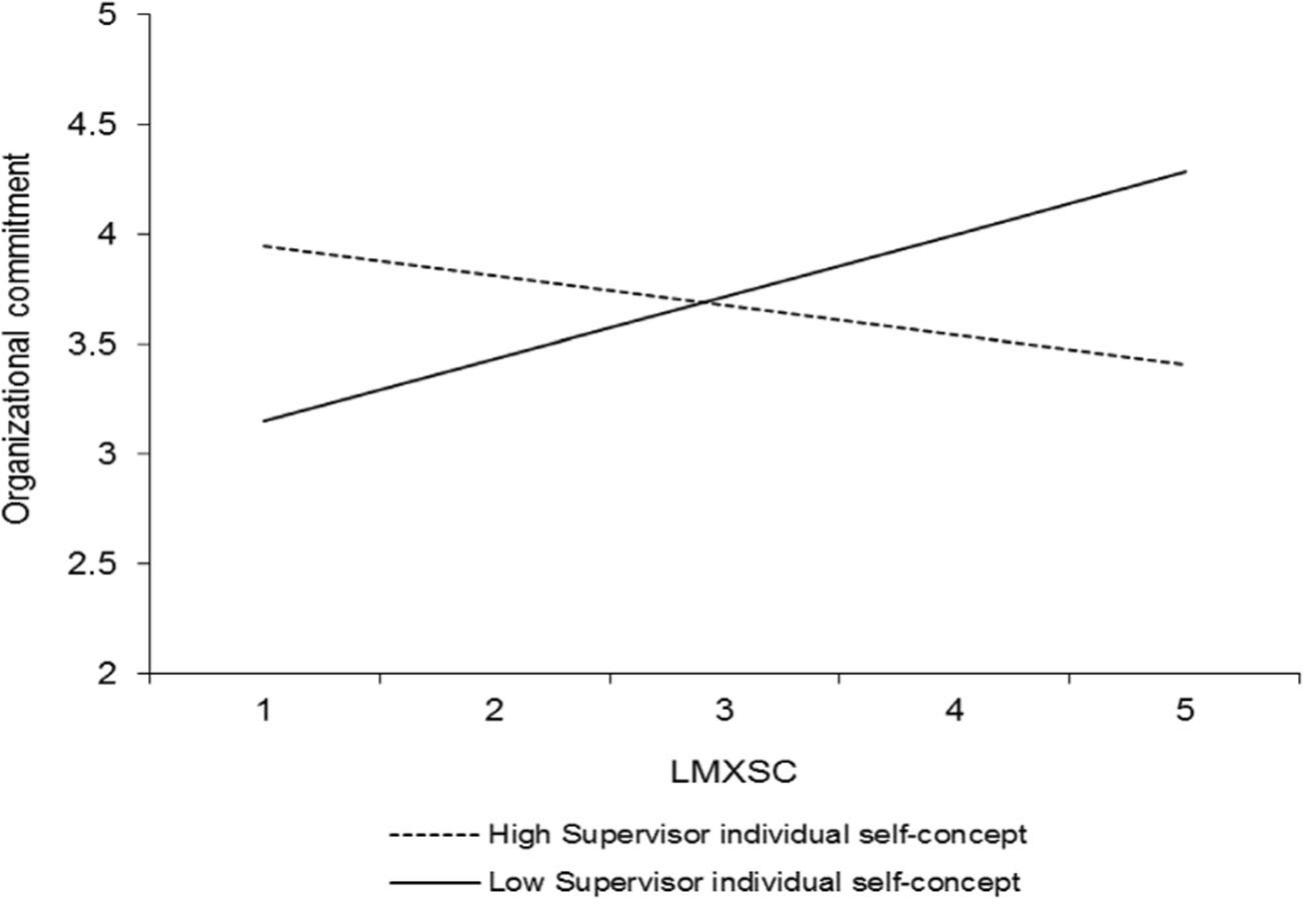
Interaction between LMXSC and perceived supervisor individual self-concept in predicting organizational commitment. Slopes are reported at 1 *SD* above and below the mean of the moderator

**Fig. 8:**
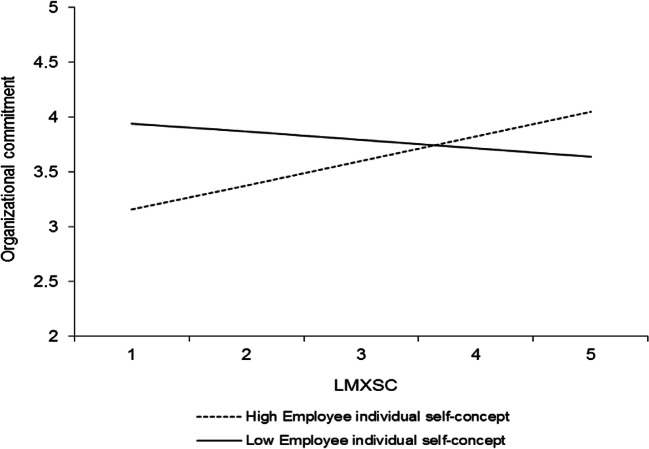
Interaction between LMXSC and employee individual self-concept in predicting organizational commitment. Slopes are reported at 1 *SD* above and below the mean of the moderator

**Fig. 9:**
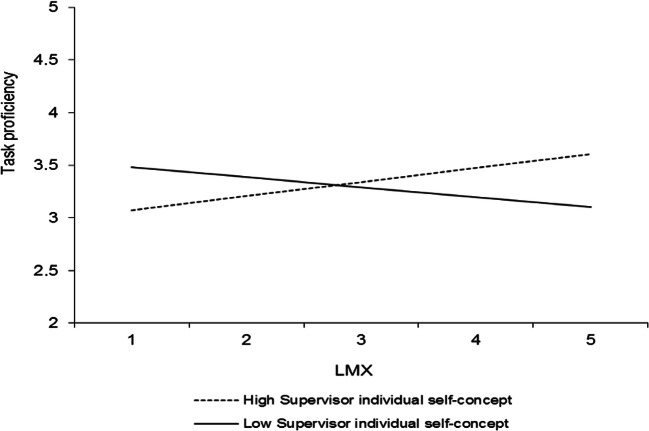
Interaction between LMX and perceived supervisor individual self-concept in predicting task proficiency. Slopes are reported at 1 *SD* above and below the mean of the moderator

In addition to the results that pertain to our hypotheses, four other interactions were significant, and all involved the individual level of the self-concept. Table 3 (model 2) shows that LMX interacted with the perceived supervisor individual self-concept to predict organizational commitment (*γ* = .20, *p* < .05). This interaction is plotted in Fig. 6. Simple slopes analyses revealed that LMX was positively related to organizational commitment at high levels (+ 1 *SD*) of the perceived supervisor individual self-concept (*γ* = .53, *t* = 4.01, *p* < .0001) but unrelated to organizational commitment at low levels (− 1 *SD*) of the perceived supervisor individual self-concept (*γ* = .14, *t* = 1.41, *ns*). Furthermore, LMXSC interacted with the perceived supervisor individual self-concept to predict organizational commitment (*γ* = − .22, *p* < .001; Table 3, model 2). This interaction is graphed in Fig. 7. LMXSC was positively related to organizational commitment when the perceived supervisor individual self-concept was low (− 1 *SD*) (*γ* = .29, *t* = 3.17, *p* < .01) but unrelated to organizational commitment when the perceived supervisor individual self-concept was high (+ 1 *SD*) (*γ* = − .15, *t* = − 1.71, *ns*). LMXSC also interacted with the employee individual self-concept to predict organizational commitment (*γ* = .17, *p* < .05; Table 3, model 2). This interaction is reported in Fig. 8. Based on simple slopes analyses, LMXSC was positively related to organizational commitment at high levels (+ 1 *SD*) of the employee individual self-concept (*γ* = .25, *t* = 2.66, *p* < .01) but unrelated to organizational commitment at low levels (− 1 *SD*) of this moderator (*γ* = − .10, *t* = − 1.10, *ns*). Finally, LMX interacted with the perceived supervisor individual self-concept to predict task proficiency (*γ* = .16, *p* < .05; Table 4, model 2). This interaction is illustrated in Fig. 9. Specifically, LMX was unrelated to task proficiency when the perceived supervisor individual self-concept was very high (+ 2 *SD*) (*γ* = .32, *t* = 1.64, *ns*) but negatively related to task proficiency when the perceived supervisor individual self-concept was very low (− 2 *SD*) (*γ* = − .31, *t* = − 2.03, *p* < .05). We elaborate on these unexpected findings in the discussion.

## Discussion

This study had two main goals, namely (a) to examine the effects of LMX compared with LMXSC on organizational commitment and, indirectly, on work role performance and (b) to examine the employees’ and perceived supervisor’s self-concept levels as moderators of these relationships. As expected, the findings showed that LMX related to performance through organizational commitment. Regarding the role played by the employees’ and perceived supervisor’s self-concept levels as contingencies, a more complex pattern of results was found. Some findings supported our hypotheses, but nonsignificant and unexpected effects also emerged.

### LMX vs. LMXSC Predicting Commitment and Performance

In this study, LMX was positively related to organizational commitment, and through it, to work role performance. These findings show that the quality of the relationship between employees and their supervisor, as captured by LMX, represents an important driver of work outcomes. They also suggest that on their own, social exchange mechanisms (Settoon, Bennett, & Liden, 1996; Wayne, Shore, & Liden, 1997) suffice to explain how the leader-member relationship relates to work outcomes, while this may not be the case for social comparison processes (Buunk & Gibbons, 2007; Festinger, 1954; Vidyarthi et al., 2010). Indeed, LMXSC did not explain variance in commitment and performance, over and above LMX. These results differ, to some extent, from the results obtained by Vidyarthi et al. (2010). These authors found LMXSC to predict performance outcomes, over and above LMX. Nevertheless, the two studies focused on different aspects of performance. Vidyarthi et al.’s study examined in-role performance and citizenship behavior directed at both other individuals within the organization (i.e., interpersonal helping) and the organization itself (i.e., loyal boosterism). Thus, the performance outcomes included in this study spanned multiple referents, some of which may plausibly bear more relevance than other referents as outcomes of social comparison processes (Buunk & Gibbons, 2007; Greenberg et al., 2007). The divergent results between this study and the present study emphasize the importance for future research to identify the outcomes to which LMXSC may be more strongly related. These differences also emphasize the importance of identifying the contexts where the social aspects of employees’ relationship with the leader (i.e., LMXSC) exert their strongest effects on attitudes and behaviors. As discussed in the next section, our study offers some insights on this issue.

### The Role of the Employee and Perceived Supervisor Self-Concept Levels

This study sheds light on the boundary conditions associated with LMX and LMXSC. We examined how the employee and perceived supervisor levels of the self-concept moderated the LMX- and LMXSC-outcome relationships, which is more inclusive than previous work that focused exclusively on the employee self-concept (e.g., Johnson et al., 2006) or a specific level of it (e.g., Chang & Johnson, 2010). The results show that the three levels of the self-concept were involved in different interactions with LMX and LMXSC. Therefore, the findings suggest that self-concept levels have relatively independent effects on individuals’ attitudes and behaviors (Brewer & Gardner, 1996; Johnson et al., 2006). Moreover, the results show different patterns of interactions for the employee and the perceived supervisor self-concept levels. This suggests that the internal attributions (Kelley & Michela, 1980) generated by employees about their supervisor’s self-concept are important, over and above employees’ own self-concept and that they should be further considered in future research.

#### Self-Concept Levels as Contingencies

First, the findings show that the employee and perceived supervisor relational self-concepts strengthen the relationship between LMXSC and commitment. This result supports our contention that the relational self-concept is more relevant in the context of LMXSC than in the context of LMX. The social comparison process that underlies LMXSC supposes that the cues of the social environment are cognitively processed to reach conclusions about whether one’s relationship with the leader is better or worse than other group members’ relationship with the leader. The relational level of the self-concept, with its focus on dyadic relationships (Brewer & Gardner, 1996; Lord et al., 1999), facilitates this process. Employees with a strong relational self-concept naturally seek and value information that pertains to their relationship with the leader, while when it is perceived as strongly defining the supervisor, the relational self-concept makes having a favorable LMX standing more rewarding. However, these moderating effects were nonsignificant at the conventional *p* < .05 level regarding the indirect relationships between LMXSC and performance. Nevertheless, most of these moderating effects (re: hypotheses 2b and 3b as related to task adaptivity and proactivity) were marginally significant (*p* < .10). This finding may indicate that although the relational self-concept contributes to shape individuals’ attitude toward the organization in response to LMXSC, it exerts, in and of itself, a weaker effect on how individuals perform in response to it.

A plausible explanation is that employee performance may be more influenced by contextual factors than job attitudes. Thus, aspects of the work environment and the job (Golden & Veiga, 2008; Judge & Zapata, 2015) should be examined in conjunction with leader-member relationships and self-concept levels in future research. Contextual factors may potentially come into play when LMXSC (vs. LMX) is combined with the relational and collective (vs. individual) levels of the self-concept. Indeed, social comparison processes, by definition, reflect and are shaped by how individuals process external cues (Buunk & Gibbons, 2007; Wood, 1996), while the relational and collective levels of the self-concept both represent a social extension of the self (Brewer & Gardner, 1996) and should therefore also be more sensitive to external influences.

Similarly, the perceived supervisor collective self-concept was found to strengthen the relationship between LMXSC and commitment. This supports our contention that when the supervisor is viewed as having a strong collective self-concept, employees experience the relationship with him or her as representing the relationship with the organization itself (Eisenberger et al., 2010). In the context of LMXSC, a strong supervisor collective self-concept makes employees feel closer to the organization and, thus, more likely to commit to it. Again, this effect did not extend to the performance dimensions (i.e., the moderated mediation effects predicted in hypothesis 5b were only marginally significant [*p* < .10]). Future research should examine how employees’ perception of their supervisor collective self-concept shapes the relationship between LMXSC and performance in conjunction with contextual factors.

Interestingly, the perceived supervisor collective self-concept was found to weaken rather than strengthen the relationship between LMX and commitment, and it also weakened the indirect LMX-performance relationships. These findings do not necessarily contradict the idea that employees view their relationship with the leader as representing the relationship with the organization when the perceived supervisor collective self-concept is high. However, these findings plausibly reflect differences in the way that this mechanism operates in the context of LMX compared with LMXSC. When employees make sense of the quality of their relationship with the leader (i.e., LMX), they assess how their supervisor treats them independently of how other employees are treated. They ask “is my relationship with my supervisor good overall?” Thus, in the context of LMX, employees who view their supervisor as having a strong collective self-concept, i.e., being driven by a concern for the welfare of the organization and its members (Brewer & Gardner, 1996), may feel they are well treated simply because they are members of the organization (i.e., in-group members). Therefore, having a high-quality LMX relationship with such leaders does not reflect interpersonal trust or individualized consideration. It may even lead employees to feel they are not sufficiently considered to be “individuals.” This interpretation suggests that LMX leads to less (rather than more) reciprocation in this context (Blau, 1964; Schopler & Thompson, 1968).

Furthermore, an intriguing set of results pertains to the moderating effects exerted by the individual level of the self-concept. First, employees’ perception of their supervisor’s self-concept seemed, again, to operate differently in the context of LMX compared with LMXSC. On the one hand, LMX significantly interacted with the perceived supervisor individual self-concept such that it was related to lower organizational commitment (Fig. 6) and task proficiency (Fig. 9) when this level of the self-concept was low (vs. high). On the other hand, LMXSC was more strongly related to organizational commitment among individuals who perceived their supervisor to have a low individual self-concept (Fig. 7). These differences may reflect the fact that LMX captures the intrinsic quality of the relationship that employees have with the leader, while LMXSC captures a contextualized view of this relationship. As mentioned previously, when employees make sense of the quality of their relationship with the leader (i.e., LMX), they assess how their supervisor treats them independently of how other employees are treated. They ask “is my relationship with my supervisor good overall?” Thus, one may speculate that LMX has more value for employees when the supervisor is perceived to focus on his or her personal welfare and success (i.e., when the supervisor is perceived to have a strong individual self-concept; Brewer & Gardner, 1996). Maybe earning a high-quality LMX relationship with such a leader is perceived to be more authentic because it involves a leader who is sparing with his or her quality relationships with subordinates.

In contrast, LMXSC may be more valuable when employees perceive that their supervisor has a low individual self-concept. A low level of the perceived supervisor individual self-concept likely reveals that the supervisor is selfless (Rus, van Knippenberg, & Wisse, 2010). Therefore, it supposes that the supervisor is seen as being more concerned with the needs of other individuals than with his or her own needs. When such a supervisor is perceived to establish a better relationship with one employee than with other employees (i.e., a high LMXSC), the focal employee should feel that his or her specific needs strongly matter to the supervisor. Because this specific consideration makes the receipt of inducements more valuable (Blau, 1964; Schopler & Thompson, 1968), employees may express more commitment in response to a high LMXSC when the supervisor is perceived to have a low individual self-concept.

Finally, the employee individual self-concept also interacted with LMXSC. LMXSC was more strongly related to organizational commitment when employees held a strong individual self-concept (Fig. 8). Because they derive their self-worth from being different (and better) than other individuals (Brewer & Gardner, 1999; Lord & Brown, 2004), individuals with a strong individual self-concept should value situations where they can comparatively look good. As LMXSC indicates the extent to which a focal employee receives better treatment from the leader than other employees receive, it makes sense that it leads to more organizational commitment among the employees with a strong individual self-concept.

#### Self-Concept Levels as Main Predictors

Self-concept levels exerted main effects on organizational commitment and performance. The employee and perceived supervisor relational and collective self-concepts were positively related to commitment, while the employee collective self-concept was positively related to task proficiency. The main effects for collective self-conceptions are consistent with previous research that reports significant relationships among the collective self-concept, organizational commitment, and performance (e.g., Johnson & Chang, 2006; Johnson et al., 2006; see also Johnson & Chang, 2008). However, the main effect of relational self-conceptions on commitment is somewhat unexpected as researchers previously claimed it should have little impact on commitment because of its dyadic focus (Johnson & Chang, 2006; Johnson et al., 2010). In our view, these findings illustrate the idea that organizational commitment is a reflection of a high-quality, social exchange-based relationship between the employee and the organization or its representatives (Eisenberger et al., 2010; van Knippenberg & Sleebos, 2006) and is thus meaningful for employees who focus on dyadic relationships as much as on collective welfare.

### Limitations

First, the data for the study were gathered at a single point in time, which limited our ability to determine the temporal ordering of the variables. However, analyses conducted on panel data from an independent sample (footnote 3) and supplementary analyses conducted on our original dataset (footnote 4) provided additional evidence that supported the ordering of the constructs specified in our theoretical model. Second, although our data were collected at one point in time, issues of common method variance are alleviated by the use of supervisor reports of work performance. In addition, most hypotheses involved interaction effects, which have been demonstrated to not be subject to common method variance effects (Siemsen, Roth, & Oliveira, 2010). Nevertheless, we recognize that our data do not allow us to test a true longitudinal mediation model, which would require that both employee reports of LMX and LMXSC and commitment and supervisor reports of employee performance be obtained at least at three points in time (Maxwell & Cole, 2007). Third, we relied on HLM to control for department-level dependencies in the data. However, the sample size at level two was rather low in this study (*n* = 7). This may have led to an underestimation of the variance attributable to departments in the relationships among variables at level one. Fourth, further inquiry is warranted to elucidate and formally test which psychological mechanisms underlie the employee and perceived supervisor self-concept levels. Finally, as related research suggests that self-conceptions are rooted in cultural influences (Cross, Bacon, & Morris, 2000), this study should be replicated with culturally diverse samples.

### Practical Implications

The findings suggest that LMX significantly predicts commitment and, through commitment, performance. Thus, employees who perceive that they have a high-quality relationship with their supervisor show increased organizational commitment and performance. Organizations should therefore direct the attention of supervisors toward the need to develop such relationships with employees. As high-quality relationships develop through a series of reciprocal exchanges (Cropanzano & Mitchell, 2005; Cropanzano et al., 2017), supervisors should seek to treat employees fairly, provide them with support and advice, and offer them interesting assignments, among other actions.

The findings also suggest that the effects of LMX and LMXSC are moderated by the employee and perceived supervisor self-concept levels. Importantly, LMXSC only predicted commitment and performance when the relevant moderators were considered. Therefore, organizations should encourage supervisors to pay attention to how employees compare themselves to other people in the group (i.e., social comparison processes) and implement human resource practices that enable supervisors to develop employees with specific self-conceptions. Employees who hold relational self-concepts may be of particular interest for organizations. This study’s findings show that a high employee relational self-concept strengthened the LMXSC-commitment relationship. Organizations should screen for relational self-concept during the employee recruitment and selection processes and seek to strengthen it through socialization practices (Chang & Johnson, 2010). Moreover, supervisors should pay attention to how employees interpret their self-conceptions. As this study’s results indicated that LMXSC related more strongly to commitment at high levels of a perceived supervisor relational self-concept, supervisors should be informed that being perceived as having a relational focus is particularly beneficial. Supervisors who detect that some employees feel frustrated from having a lower LMX than other employees (i.e., a low LMXSC) may also want to signal the importance that they attribute to dyadic relationships with employees (e.g., by offering consistent feedback over time or taking the time to address employees’ concerns on a regular basis), as this would foster perceptions of a relational self-concept. Emphasizing relational self-conceptions among employees and supervisors seems to be even more important knowing that the employee and perceived supervisor relational self-concepts exerted positive main effects on commitment.

Moreover, although unexpected, the individual self-concept played a significant role in this study. For instance, the LMXSC-commitment relationship was stronger among employees with a strong individual self-concept. Supervisors should therefore be made aware that employees with an individual self-concept are sensitive to social comparisons in leader-member relationships. Providing cues that signal to these employees that their relationship with the supervisor is unique may intensify the effect of LMXSC. At the same time, social comparisons in leader-member relationships would be more beneficial when the supervisor does not provide cues that he or she has an individual self-concept. Indeed, we found that the relationship of LMXSC to organizational commitment was stronger when the perceived supervisor individual self-concept was low. Finally, it is important to note that not all effects that involve organizational commitment extended to the work performance dimensions in this study. Therefore, organizations should be informed that the influence of self-concept levels on performance may be more limited than the self-concept’s influence on commitment; accordingly, organizations should consider additional, context-based approaches to support employee performance.

## Footnotes

1 Note that given its perceptual nature, LMXSC more closely reflects social comparison processes than relative leader-member exchange (RLMX; Tse, Lam, Lawrence, & Huang, 2013).

2 Note that Bauer, Preacher, and Gil (2006) provide procedures for testing the significance of indirect effects in the context of multilevel models. Their method uses the asymptotic covariance matrix of the variance component estimates for the random effects (see Preacher & Hayes, 2008). However, in our HLM analyses, there were no random slopes; thus, the matrix of these slopes was null. Therefore, we used Hayes's (2013) PROCESS macro as an alternative method to test the significance of the indirect effects.

3 One weakness of our design was that all variables were measured at the same time. This may raise concerns about the causal ordering of the constructs in our theoretical model. To address this issue, we conducted a separate, panel study among a sample of 306 employees (response rate = 82%) who worked in a manufacturing company in Shandong Province, China. LMX, LMXSC, and organizational commitment were measured twice at a time interval of 5 weeks using the same scales as the scales used in our main study, except that the items were translated into Chinese through a standard translation-back-translation procedure. All measures displayed good internal consistency at both time 1 and time 2 (LMX: *α*s = .89 and .89; LMXSC: *α*s = .92 and .88; organizational commitment: *α*s = .88 and .86). Among the participants, 67% were male, average age was 36.44 years (*SD* = 7.04), average organizational tenure was 13.72 years (*SD* = 6.67), and average tenure with the supervisor was 7.02 years (*SD* = 3.31). We used LISREL 8.80 (Jöreskog et al., 2001) to examine a cross-lagged panel model of the temporal relationships among our variables. To simplify the model, we averaged the items per dimension of LMX to create four aggregate indicators for the construct at time 1 and time 2. The other constructs were examined at the item level. As the constructs were moderately correlated, we allowed the latent variables’ residuals to be correlated at time 2. The cross-lagged model included stability effects and cross-lagged effects among the variables from time 1 to time 2. This model yielded a good fit to the data, *χ*^2^ (449) = 631.54, *p* < .001, RMSEA = .035, CFI = .99, TLI = .98, SRMR = .043. In accounting for the stability effects, time 1 LMXSC and time 1 LMX exerted a positive effect on time 2 organizational commitment (*γ* = .50, *p* < .001, and *γ* = .96, *p* < .01, respectively), while time 1 organizational commitment exerted no effect on time 2 LMXSC and time 2 LMX (γ = −.04, *ns*, and *γ* = .08, *ns*, respectively). These results suggest that LMXSC and LMX temporally precede organizational commitment.

4 As an additional analysis of our original data, we used structural equation modeling in LISREL 8.80 (Jöreskog et al., 2001) to examine two competing orderings of our variables, namely (a) LMX-LMXSC → organizational commitment → work performance and (b) work performance → LMX-LMXSC → organizational commitment. In each model, we averaged the items per dimension of LMX to create four indicators, while the other constructs were examined at the item level. In model (a), we allowed the residuals of task proficiency and task adaptivity and proactivity to be correlated because the constructs were moderately correlated. In absolute terms, the fit indices associated with model (b) were poorer (*χ*^2^ (268) = 889.11, *p* < .001, RMSEA = .110, CFI = .95, TLI = .94, SRMR = .110) than the fit indices for model (a) (*χ*^2^ (269) = 843.75, *p* < .001, RMSEA = .100, CFI = .95, TLI = .94, SRMR = .072). Moreover, in model (a), organizational commitment was significantly related to task proficiency (*γ* = .34, *p* < .001) and task adaptivity and proactivity (*γ* = .37, *p* < .001), while in model (b), task proficiency and task adaptivity and proactivity were unrelated to LMXSC (*γ* = .06, *ns*, and *γ* = .13, *ns*, respectively); only task adaptivity and proactivity, not task proficiency, was significantly related to LMX (*γ* = .36, *p* < .001, and *γ* = − .07, *ns*, respectively). These findings indicate that the work performance dimensions weakly explain LMX/LMXSC and that model (a) is a better representation of the relationships among the variables.

5 We initially used Bauer et al.’s (2006) program, which is suitable to test moderated mediation in the context of multilevel models. However, the program did not converge for any of the moderated mediation models, which is not uncommon (e.g., Hannah et al., 2013).

6 Note that the results that pertain to the hypotheses remained significant and virtually the same when the controls were dropped from the models.
